# Supplementary material for: Identification of a Novel p53 Modulator Endowed with Antitumoural and Antibacterial Activity through a Scaffold Repurposing Approach
Source: Pharmaceuticals (Basel). 2022 Oct 26;15(11):1318. doi: 10.3390/ph15111318 (PMC9692514; doi:10.3390/ph15111318)
Supplement: Supplementary file 1 [file pharmaceuticals-15-01318-s001.zip › pharmaceuticals-1955303-supplementary.pdf]

SUPPLEMENTARY INFORMATION

Identification of a novel p53 modulator endowed with antitumoral and antibacterial activity through a scaffold repurposing approach

Elisa Nuti[1], Valeria La Pietra[2], Simona Daniele[1], Doretta Cuffaro[1], Lidia Ciccone,[1] Chiara Giacomelli[1], Carolina Cason[3], Alfonso Carotenuto[2], Vincenzo Maria D'Amore[2], Eleonora Da Pozzo[1], Barbara Costa[1], Riccardo Di Leo[1], Manola Comar[3], Luciana Marinelli[2], Claudia Martini[1], Armando Rossello[1]*

[1] Department of Pharmacy, University of Pisa, via Bonanno 6, 56126 Pisa, Italy.

[2] Department of Pharmacy, University of Naples Federico II, Via D. Montesano 49, 80131 Naples, Italy

[3] Unit of Advanced Microbiology Diagnosis and Translational Research, Institute for Maternal and Child Health-IRCCS "Burlo Garofolo", Trieste, Italy.

Correspondence: Armando Rossello armando.rossello@unipi.it

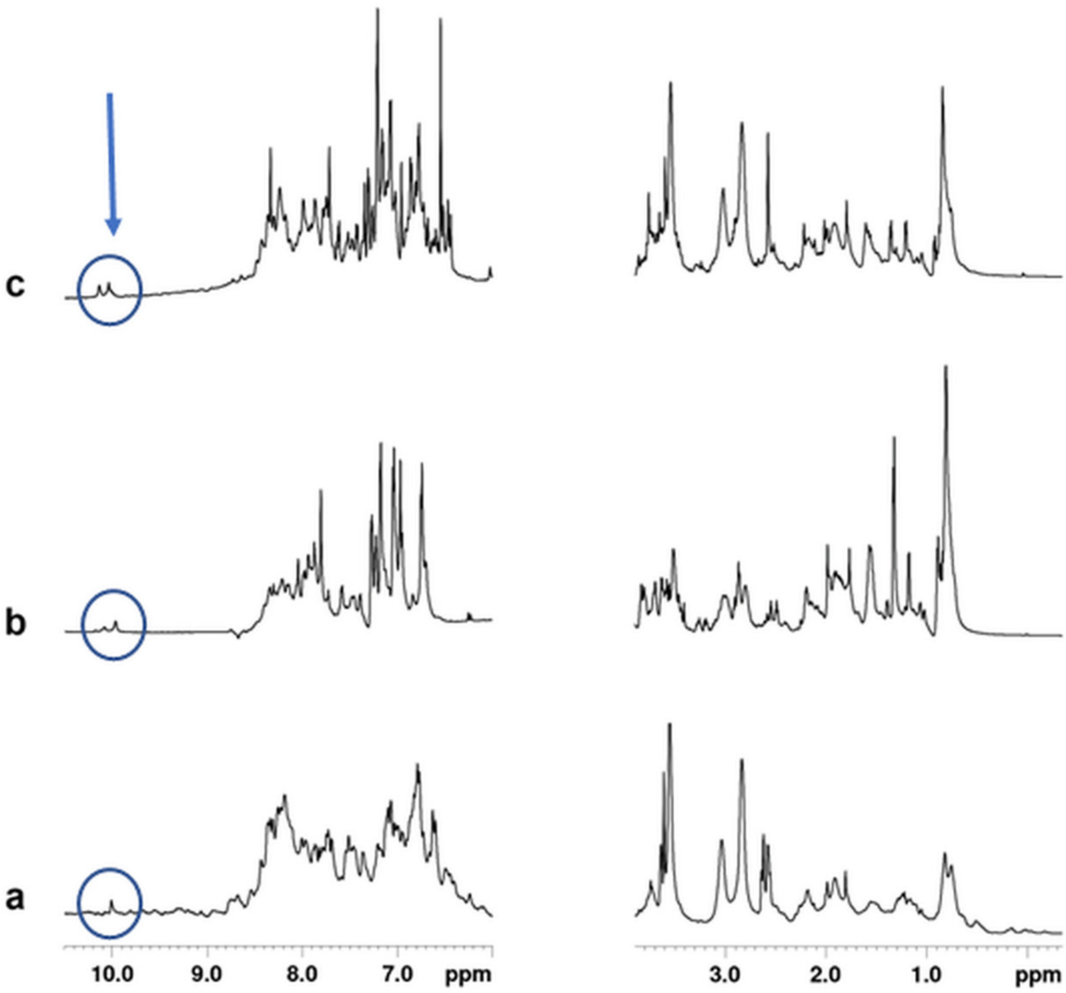

Figure S1: One-dimensional proton spectrum of the p53-MDM2 complex (**a**), p53-MDM2 complex after addition of RM37 (**b**), or Nutlin-3a (**c**). In blue are highlighted the tryptophan residues of MDM2-p53 complex, W53 and W23 $^{N}H^{\varepsilon}$ side chains signals, discussed in Figure 3.

Chemistry

General

Melting points were determined with a Kofler hot-stage apparatus and are uncorrected. ^{1}H NMR spectra were recorded in appropriate solvents with a Bruker Avance III HD 400 spectrometer operating at 400 MHz. ^{13}C NMR spectra were recorded with the above spectrometer operating at 100.57 MHz. The assignments were made, when possible, with the aid of DEPT, COSY, HSQC experiments. The first order proton chemical shifts (δ) are referenced to residual solvents and *J*-values are given in Hz. All reactions were followed by TLC on Kieselgel 60 F254 with detection by UV light and/or with ethanolic 10% phosphomolybdic or sulfuric acid, and heating. Kieselgel 60 (Merck,

230-400 mesh) was used for flash chromatography. Some chromatographic separations were conducted by using the automated system Isolera® Prime (Biotage), equipped with UV detector with variable wavelength (200-400 nm) or using prepacked ISOLUTE Flash Si II cartridges (Biotage). All reactions involving air- or moisture-sensitive reagents were performed under an argon or nitrogen atmosphere using anhydrous solvents. Anhydrous dimethylformamide (DMF), dichloromethane (CH_2Cl_2) and THF were purchased from Sigma-Aldrich. Na_2SO_4 was used as the drying agents for solutions. Elemental analysis has been used to determine the purity of target compounds. Analytical results are within ± 0.40% of the theoretical values.

Synthesis of RM37 and RM53

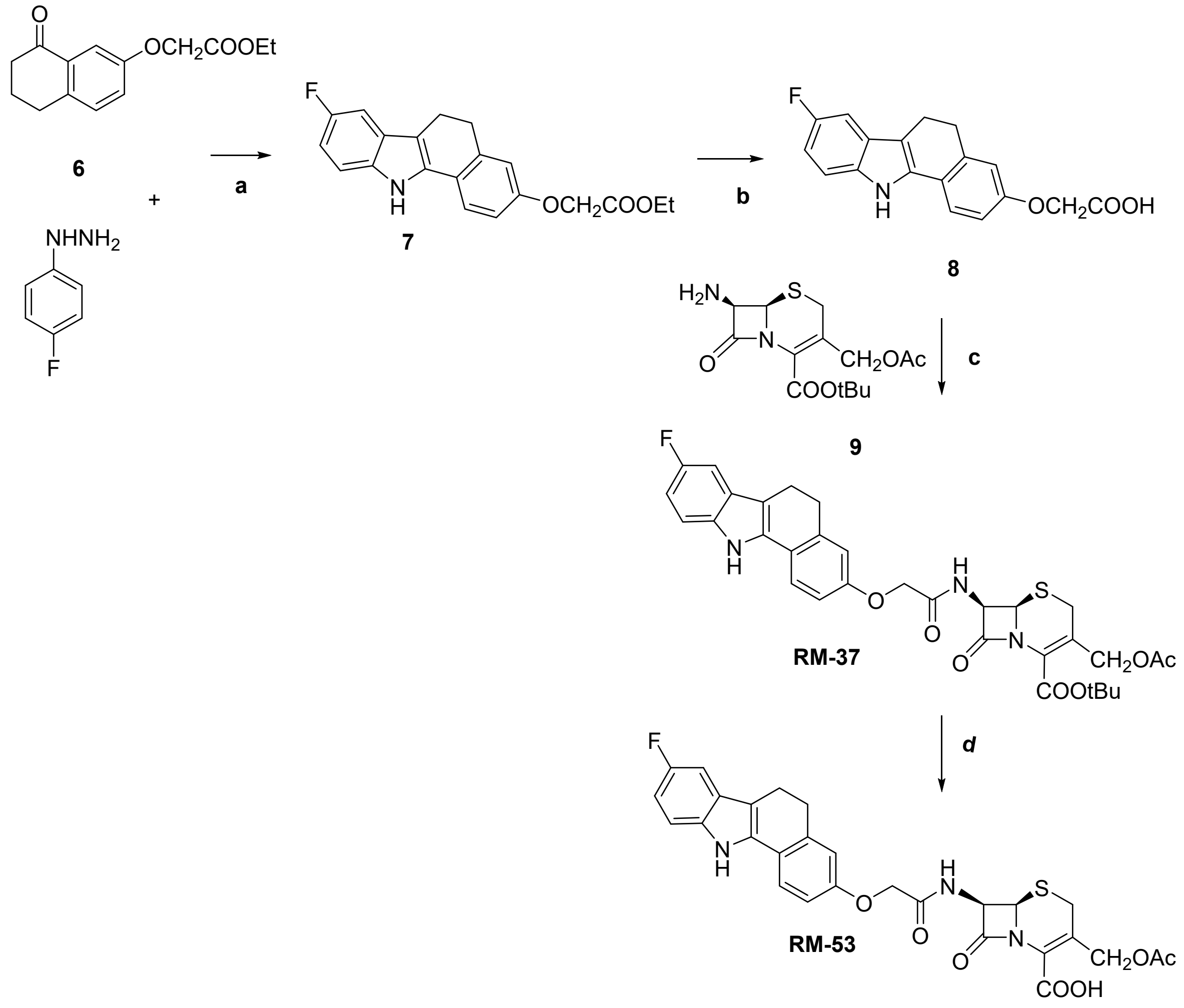

Scheme S1. Reagents and conditions[1]: a) AcOH, 120 °C; b) KOH/EtOH; c) EDC, THF, 0 °C; d) TFA, anisole, CH_2Cl_2, 0 °C.

Synthesis of ethyl 2-[(8-fluoro-6,11-dihydro-5H-benzo[a]carbazol-3-yl)oxy]acetate (7). A solution of already reported tetralone **6**[2] (2 g, 7.59 mmol) and commercial (4-fluorophenyl)hydrazine (1.28 g,

7.89 mmol) in glacial AcOH (10.6 mL) was refluxed for 6 h at 120 °C under inert atmosphere (N_2). The resulting suspension was cooled at room temperature and evaporated to give a yellow solid residue. The residue was dissolved in EtOAc (250 mL), washed with H_2O (3 x 250 mL) and $NaHCO_3$ s.s (1 x 250 mL), dried over Na_2SO_4 and evaporated under reduced pressure. The crude yellowish solid was purified by trituration with *n*-hexane/Et_2O to afford **7** as yellow solid (2.32 g, 90% yield). ^{1}H-NMR (400 MHz, $CDCl_3$) δ: 1.32 (t, *J* = 7.2 Hz, 3H); 2.83-3.06 (m, 4H); 4.31 (q, *J* = 7.2 Hz, 2H); 4.72 (s, 2H); 6.74-6.79 (m, 6H); 7.15-7.18 (m, 1H); 7.26-7.34 (m, 2H); 8.18 (brs, 1H).

*Synthesis of 2-[(8-fluoro-6,11-dihydro-5H-benzo[a]carbazol-3-yl)oxy] acetic acid (**8**).* A mixture of carbazole **7** (2.32g, 6.83 mmol) and KOH (0.460 g, 8.20 mmol) in absolute EtOH (150 mL) was stirred at room temperature for 48 h. Then the solvent was evaporated and the resulting yellow residue was dissolved in H_2O and extracted with EtOAc (2 x 125 mL). The aqueous solution was acidified with 10% HCl up to pH 1 and extracted with EtOAc (4 x 125 mL). The second extraction organic phases were collected, dried over Na_2SO_4 and evaporated to afford **8** as yellowish solid (2.17 g, quantitative yield). ^{1}H-NMR (400 MHz, DMSO-d_6) δ: 2.79-2.87 (m, 2H); 2.91-3.00 (m, 2H); 4.70 (s, 2H); 6.80-6.84 (m, 1H); 6.86-6.96 (m, 2H); 7.17-7.23 (m, 1H); 7.26-7.38 (m, 1H); 7.52-7.58 (m, 1H); 11.42 (s, 1H).

*Synthesis of (6R, 7R)-tert-butyl-7-(2-(8-fluoro-6,11-dihydro-5H-benzo[a]carbazol-3-yloxy)acetamido)-3-(acetoxymethyl)-8-oxo-5-thia-1-azabicyclo[4.2.0]oct-2-ene-2-carboxylate (**RM37**).* To a solution of the carboxylic acid **8** (0.200 g, 0.642 mmol), in anhydrous THF (31 mL), *tert*-butyl ester **9**[2] (0.211 g, 0.642 mmol) was added. The reaction mixture was cooled in an ice bath and EDC (0.136 g, 0.642 mmol) was added portionwise. The reaction was stirred under N_2 atmosphere at room temperature for 24 h. Then, the solvent was evaporated at room temperature, the residue was dissolved in $CHCl_3$ (60 mL), washed with H_2O (4 x 25 mL) The organic phase was dried over Na_2SO_4 and evaporated at 20°C affording a crude yellowish solid. The crude (405 mg) was purified by flash chromatography (*n*-hexane/EtOAc 3:1) using an Isolute column (Si II) cartridge to yield RM37 as a white solid (0.267 g, 67% yield). m.p.:104-109°C ^{1}H-NMR (400 MHz, $CDCl_3$) δ: 1.54 (s, 9H, tBu); 2.07 (s, 3H, CH_3CO); 2.90 (t, *J*= 8 Hz, 2H, -C-*CH_2*-CH_2-C); 3.03 (t, *J*= 8 Hz, 2H, -C-CH_2-*CH_2*-C); 3.32, 3.52 (2d, *J* = 18.4 Hz, 2H, -S-*CH_2*-C); 4.56 (s, 2H, -O*CH_2*-C-); 4.80 (d, *J*= 12.8 Hz, 1H, -S-*CH*-); 4.98-5.06 (m, 2H, -O-*CH_2*-CO); 5.91 (dd, *J* = 4.8 Hz, J=9.2 Hz, 1H, NH-*CH*-); 6.76 (dd, *J*= 2.8 Hz, *J*= 8.4 Hz, 1H, Ar), 6.85-6.91 (m, 2H, Ar); 7.14 (dd, *J*=2.4 Hz, 1H, Ar); 7.24-7.31 (m, 3H, Ar, NHCO); 8.36 (s, 1H, *NH*). ^{13}C NMR (100 MHz, $CDCl_3$) δ:19.6, 20.9, 26.9; 28.0, 29.9, 57.2; 58.6; 59.6; 63.2; 65.6, 67.3, 84.2, 103.6 (d, $J_{2C\text{-}F}$= 23 Hz); 110.3 (d, $J_{2C\text{-}F}$= 26 Hz); 111.5-111.7 (d, $J_{4C\text{-}F}$= 5Hz); 115.8; 117.1, 123.4 (d, $J_{3C\text{-}F}$= 12 Hz); 127.6; 127.9 (d, $J_{3C\text{-}F}$= 12 Hz); 133.4;

134.6; 139.1, 156.1; 158.0 (d, J_{1C-F}= 234 Hz); 160.4, 163.9; 168.8; 170.8; Elemental analysis calcd (%) for $C_{32}H_{32}FN_3O_7S$: C, 61.82; H, 5.19; N, 6.76; found C, 61.86; H, 5.21; N, 6.78.

*Synthesis of (6R,7R)-7-(2-[(8-fluoro-6,11-dihydro-5H-benzo[a]carbazol-3-yl)oxy]acetamido)-3-(acetoxymethyl)-8-oxo-5-thia-1-azabicyclo[4.2.0]oct-2-ene-2-carboxylic acid (**RM-53**).* Anisole (0.05 mL) and TFA (0.42 mL) were added to a cooled and stirred solution of compound **RM37** (60 mg, 0.097 mmol) in anhydrous CH_2Cl_2 (1.0 mL). The reaction mixture was stirred at room temperature, under nitrogen atmosphere, for 18 h and then evaporated at room temperature. The resulting yellow solid was triturated with Et_2O to afford compound **RM53** as white solid (18 mg, 33%, yield); m.p.:195-198°C. ^{1}H-NMR (400 MHz, DMSO-d_6) δ: 2.04 (s, 3H, CH_3CO); 2.83-2.87 (m, 2H, -C-*CH2*-CH2-C); 2.95-3.0 (m, 2H, -C-CH2-*CH2*-C); 3.48-3.69 (2d, *J* = 18 Hz, 2H, -S-*CH2*-C); 4.68-4.73 (m, 3H, 1x O-*CH2*-CO, 1x O-*CH2*-C, S-*CH*-N-); 5.01 (d, *J* = 12.8 Hz, 1H, 1x O-*CH2*-C); 5.15 (d, *J* = 4.8 Hz, 1H, 1x O-*CH2*-CO); 5.76 (dd, *J* = 4.8 Hz, J= 8.4 Hz, 1H, NH-*CH*); 6.87-6.93 (m, 3H, Ar, NH), 7.31-7.20 (m, 3H, Ar); 7.33 (q, *J*=4.4Hz, 1H, Ar); 7.57 (d, J= 9.5Hz, 1H, Ar); 9.16 (d, *J* = 8.4 Hz, 1H, -N*H*CO); 11.44 (s, 1H, NH). ^{13}C NMR (100 MHz, $CDCl_3$) δ:19.6, 21.0, 26.1; 29.7, 57.8; 59.2; 63.2; 66.7; 103.2(d, J_{2C-F}= 23 Hz); 109.2 (d, J_{2C-F}= 26 Hz); 109.6 (d, J_{4C-F}= 5 Hz); 112.3 (d, J_{3C-F}= 9 Hz); 112.9; 115.5; 122.7; 122.8; 123.8; 125.8; 127.0; 127.5 (d, J_{3C-F}= 10 Hz); 128.7; 129.4; 133.9; 135.6; 138.5; 157.3; 157.6 (d, J_{1C-F}= 230 Hz); 163.3; 164.6; 169.1; 170.7; Elemental analysis calcd (%) for $C_{28}H_{24}FN_3O_7S$: C, 59.49; H, 4.28, N, 7.43; found C, 59.51; H, 4.31; N, 7.46.

RM-37 ^{1}H NMR ($CDCl_3$ 400MHz):

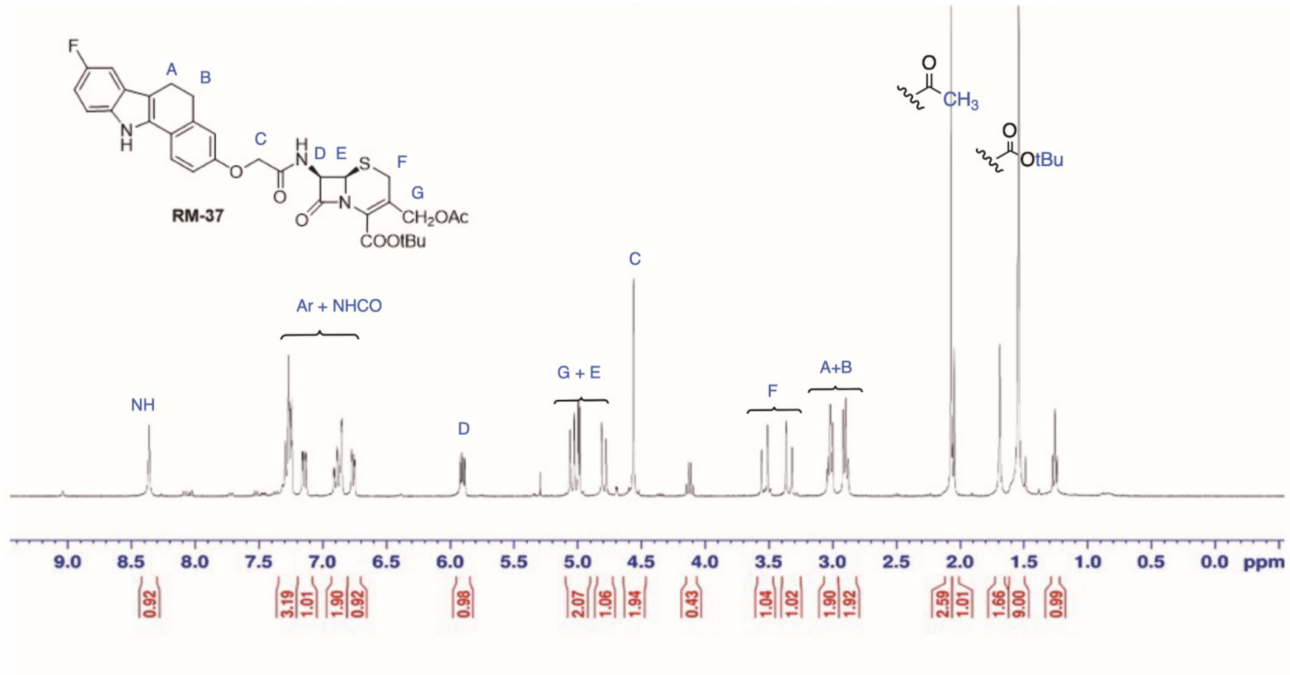

RM-37 ^{13}C NMR ($CDCl_3$ 100MHz):

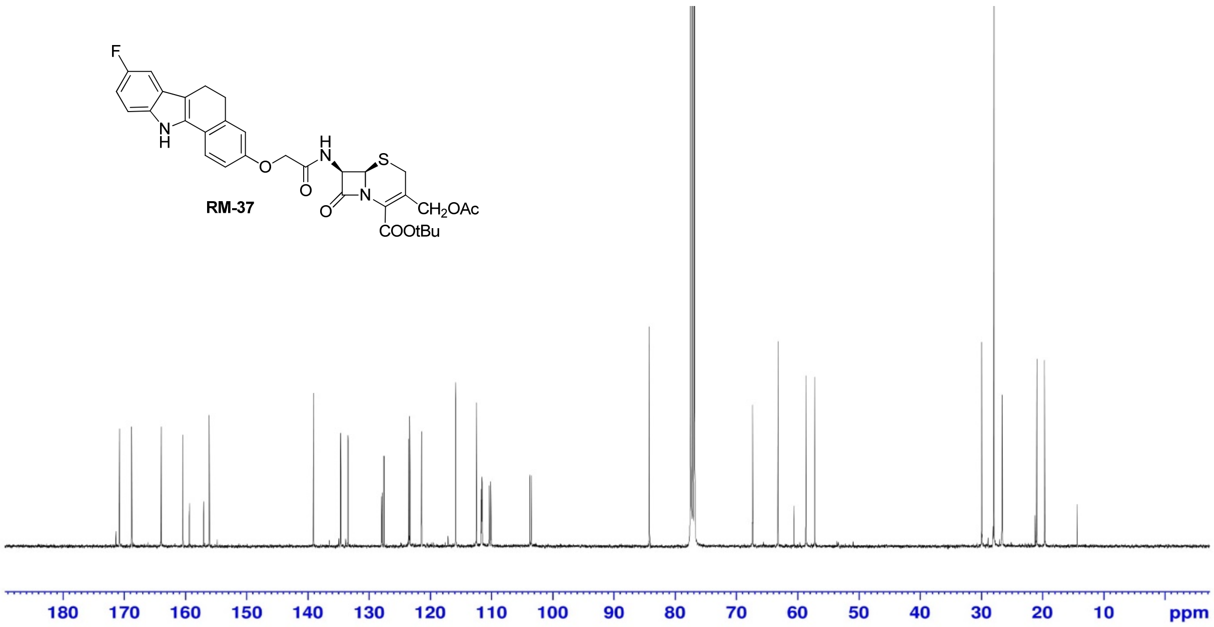

RM-37 HSQC ($CDCl_3$ 100MHz):

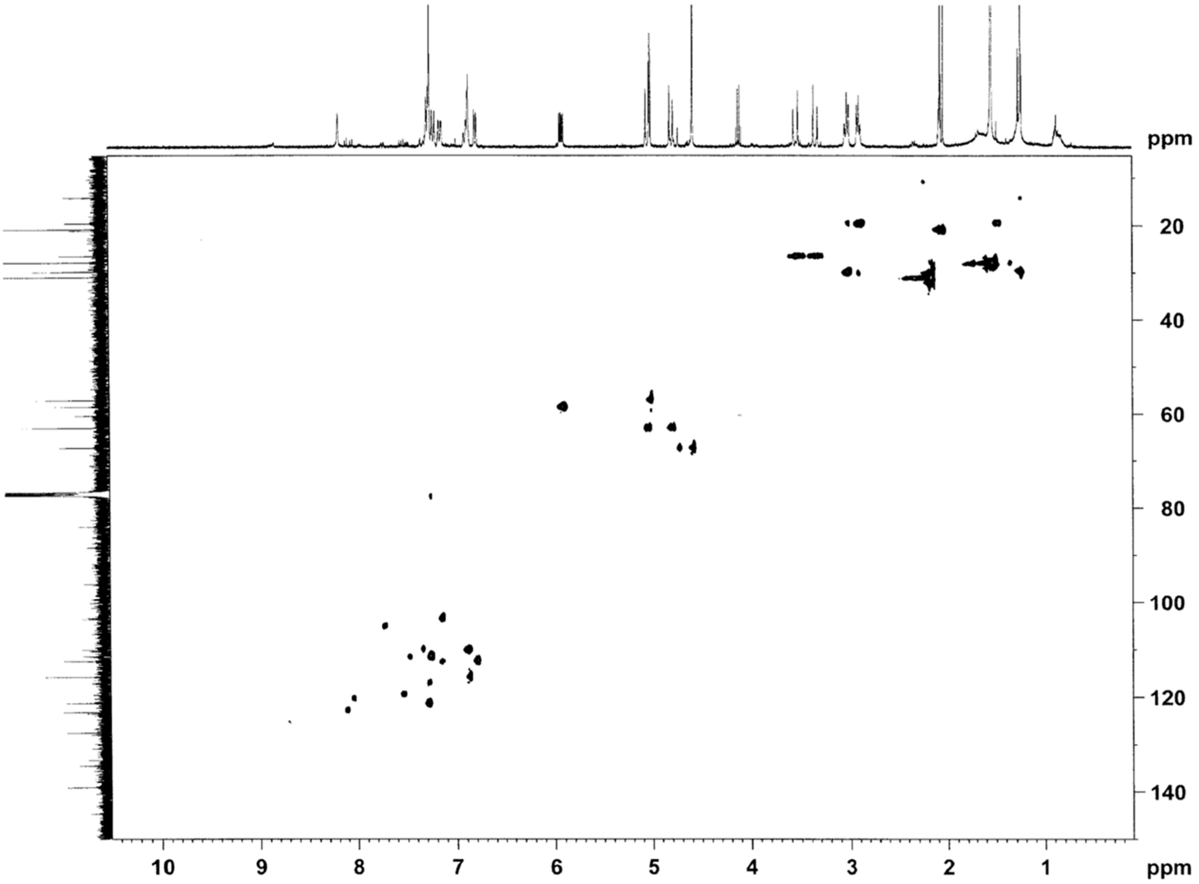

RM-53 ^{1}H NMR (DMSO-d_6 400MHz):

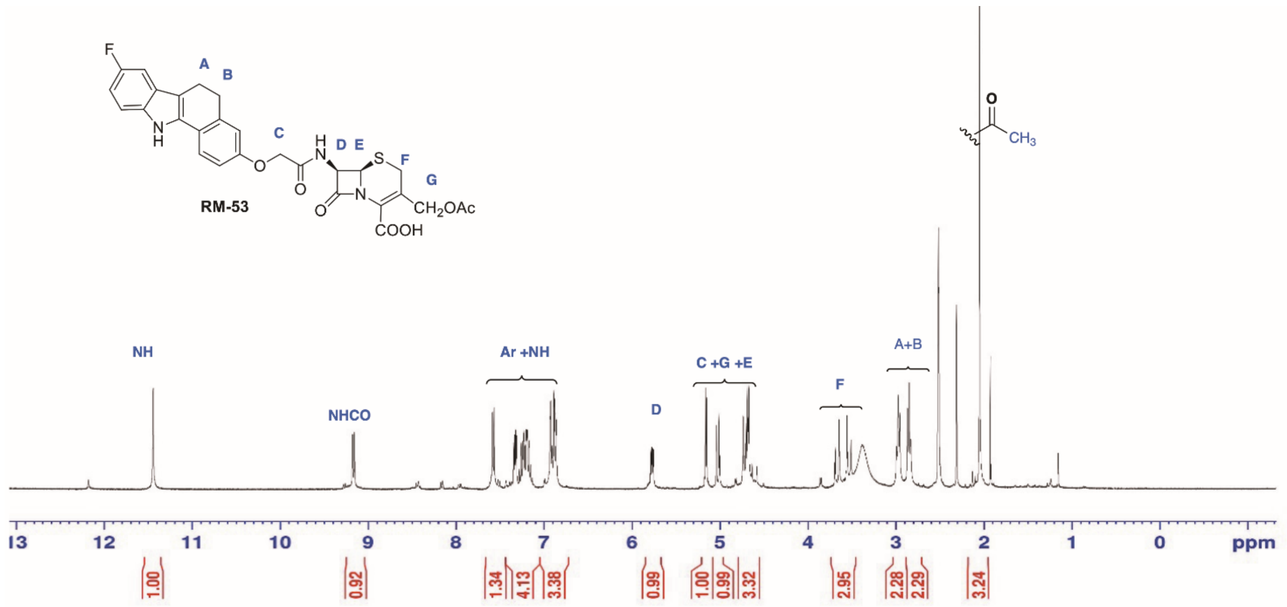

RM-53 ^{13}C NMR (DMSO-d_6 100MHz):

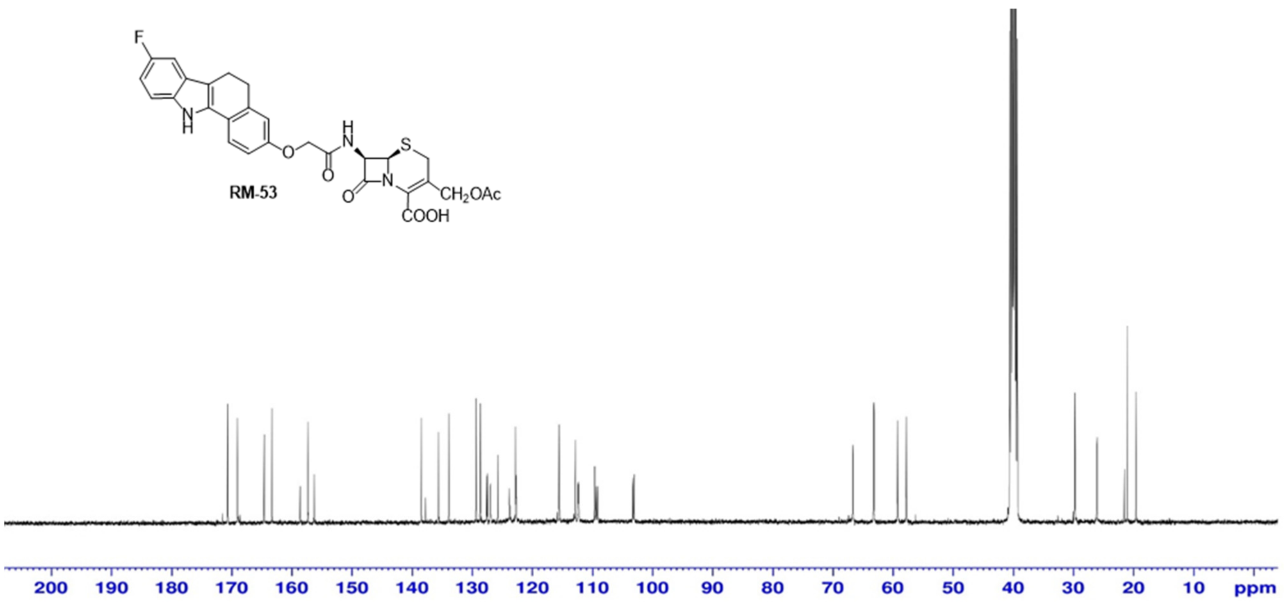

---

[1] Rossello, A. et al. Patent WO 2019/049024 Al, **2019**.
[2] Rossello, A. et al. Synthesis and antimicrobial activity of new 7 beta-(benzo[a]dihydrocarbazolyloxyacetyl)-substituted cephalosporins. *Farmaco,* **2004**, *59*, 691-696. doi:10.1016/j.farmac.2004.05.001
